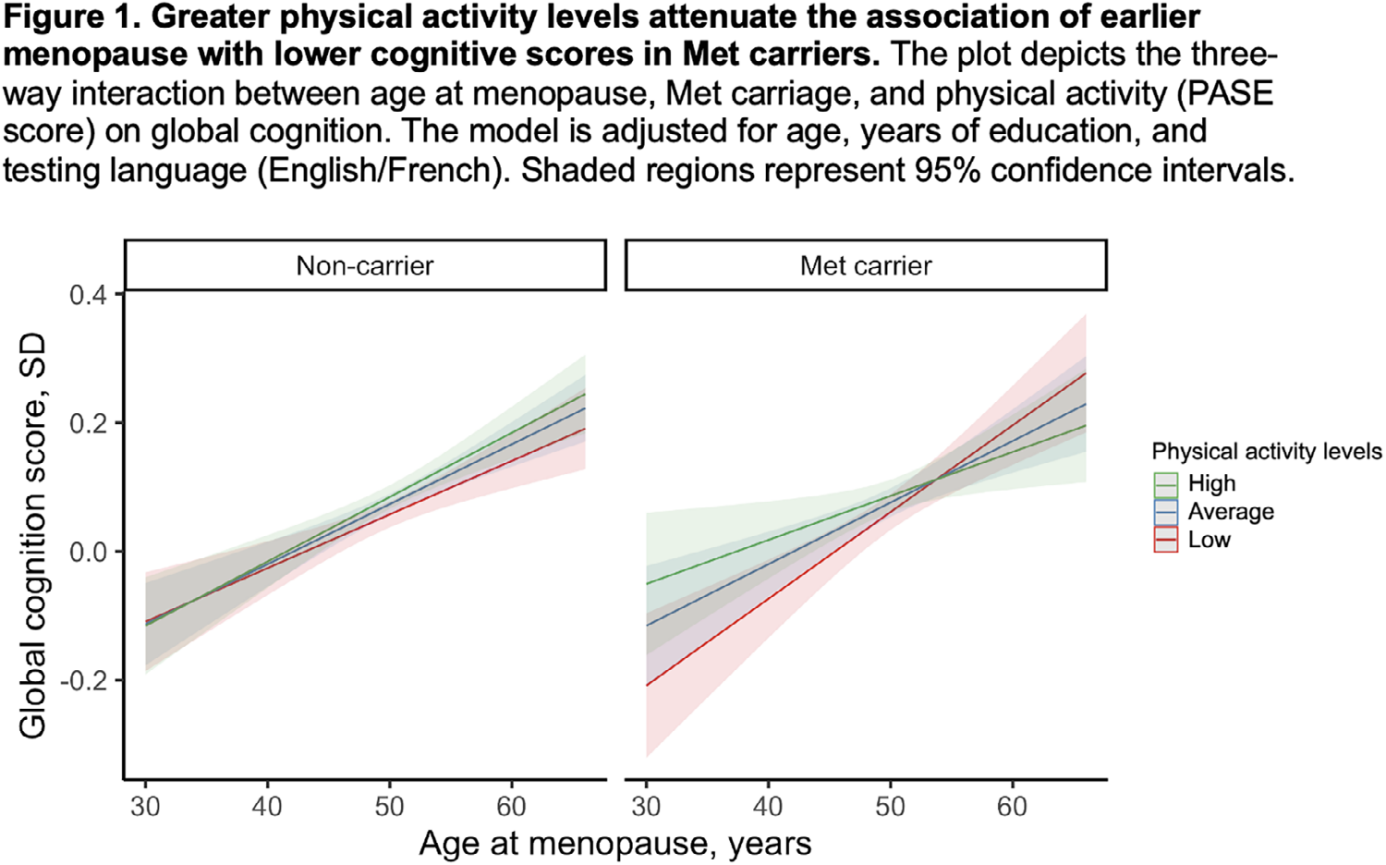# BDNF Val66Met genotype, age at menopause, and physical activity synergistically influence cognition in postmenopausal women

**DOI:** 10.1002/alz70860_105084

**Published:** 2025-12-23

**Authors:** Madeline Wood Alexander, Jane Paterson, Yuen Yan Wong, Walter Swardfager, Sandra E. Black, Jennifer S Rabin

**Affiliations:** ^1^ University of Toronto, Toronto, ON, Canada; ^2^ Sunnybrook Research Institute, Toronto, ON, Canada; ^3^ Hurvitz Brain Sciences Program, Sunnybrook Research Institute, Toronto, ON, Canada; ^4^ Department of Pharmacology and Toxicology, University of Toronto, Toronto, ON, Canada; ^5^ Hurvitz Brain Sciences Program, Toronto, ON, Canada; ^6^ Division of Neurology, Department of Medicine, University of Toronto, Toronto, ON, Canada; ^7^ Dr. Sandra Black Centre for Brain Resilience & Recovery, Toronto, ON, Canada

## Abstract

**Background:**

Earlier age at menopause is a risk factor for cognitive decline and dementia. Carriers of the Met allele of the Val66Met polymorphism show decreased levels of brain‐derived neurotrophic factor (BDNF), which is associated with worse cognitive outcomes. Previous research suggests that higher BDNF levels may help mitigate the cognitive deficits associated with estrogen depletion in postmenopausal women. Physical activity (PA) increases BDNF levels and might further counteract the effects of earlier menopause and/or Met allele carriage on cognitive outcomes. Despite these known associations, the combined influence of these factors remains unknown. Here, we investigated whether BDNF genotype, age at menopause, and PA interact to influence cognition in postmenopausal women.

**Method:**

We used baseline data from the Canadian Longitudinal Study on Aging. BDNF Val66Met genotype was dichotomized as Met carriers (Met/Met or Met/Val) vs. non‐carriers (Val/Val). Age at menopause was self‐reported. PA was measured using the Physical Activity Scale for the Elderly (PASE) score. Global cognition was assessed with neuropsychological tests and quantified with a composite of standardized scores. Linear regression models were used to test independent and interactive associations of age at menopause, Met carriage, and PASE score on cognitive scores, adjusting for age, years of education, and testing language (English/French).

**Results:**

We included *N* = 8,101 postmenopausal women (mean age=64.5±8.78 years, mean age at menopause=50.0±4.86 years, 33% Met carriers). In terms of independent effects, earlier menopause and lower PA were each associated with worse cognition (age at menopause: *β* = 0.070, *p* < .001; PA: *β* = 0.033, *p* = .001). However, Met carriage alone was not associated with cognition (*β* = 0.005, *p* = .81). Notably, there was a significant three‐way interaction between age at menopause, Met carriage, and PA on cognition. Specifically, greater PA levels attenuated the influence of earlier menopause on worse cognition in Met carriers, but not in non‐carriers (Figure 1; *β* = ‐0.048, *p* = .03). Sensitivity analysis adjusting for hormone therapy and vascular risk yielded similar results.

**Conclusion:**

These findings suggest that age at menopause, BDNF genotype, and PA synergistically influence cognition in postmenopausal women. Staying physically active may be particularly beneficial for cognitive health in women with the Met allele and an earlier age at menopause.